# A Well-Circumscribed Border with Peripheral Doppler Signal in Sonographic Image Distinguishes Epithelioid Trophoblastic Tumor from Other Gestational Trophoblastic Neoplasms

**DOI:** 10.1371/journal.pone.0112618

**Published:** 2014-11-14

**Authors:** Jiale Qin, Weiwen Ying, Xiaodong Cheng, Xiaodong Wu, Bingjian Lu, Yun Liang, Xinyu Wang, Xiaoyun Wan, Xing Xie, Weiguo Lu

**Affiliations:** 1 Department of Ultrasound, Women's Hospital, School of Medicine, Zhejiang University, Zhejiang, China; 2 Department of Women's Health, Women's Hospital, School of Medicine, Zhejiang University, Zhejiang, China; 3 Department of Oncology, Women's Hospital, School of Medicine, Zhejiang University, Zhejiang, China; 4 Department of Pathology, Women's Hospital, School of Medicine, Zhejiang University, Zhejiang, China; Chinese Academy of Sciences, China

## Abstract

As epithelioid trophoblastic tumor (ETT) shares similar clinical features with other gestational trophoblastic neoplasms (GTNs), it is likely to be clinically misdiagnosed and subsequently treated in an improper way. This study aimed to identify the sonographic features of ETT that are distinct from other GTNs, including placental site trophoblastic tumor (PSTT) and invasive mole/choriocarcinoma (IM/CC). Here, we retrospectively analyzed ultrasound images of 12 patients with ETT in comparison with those of 21 patients with PSTT and 24 patients with IM/CC. The results showed that maximal diameter and hemodynamic parameters were not significantly different among ETT, PSTT and IM/CC (P>0.05). However, a well-circumscribed border with hypoechogenic halo was identified in the gray-scale sonogram in all 12 cases of ETT, while only in 1 out of 21 cases of PSTT and 1 out of 16 cases of IM/CC (P<0.001 for ETT vs. PSTT or IM/CC). Moreover, a peripheral pattern of Doppler signals was observed in 11 out of 12 ETT lesions, showing relatively more Doppler signal spots around the tumor border than within the boundary, while a non-peripheral pattern of Doppler signals in all 21 PSTT cases and 14 out of 16 IM/CC cases: with minimal, moderate or remarkable signal spots within the tumor, but not along the tumor (P<0.001 for ETT vs. PSTT or IM/CC). These distinct sonographic features of ETT correlated with histopathologic observations, such as expansive growth pattern and vascular morphology. Thus, we draw the conclusions that the well-circumscribed border with peripheral Doppler signal may serve as a reliable sonographic feature to discriminate ETT from other types of GTNs. With further validation in a larger patient set in our ongoing multi-center study, this finding will be potentially developed into a non-invasive pre-operative GTN subtyping method for ETT.

## Introduction

Gestational trophoblastic neoplasm (GTN) is clinicopathologically categorized into invasive mole (IM), choriocarcinoma (CC), placental site trophoblastic tumor (PSTT) and epithelioid trophoblastic tumor (ETT). ETT has only recently been identified and classified as an entity distinct from other types of GTNs [1]. It has an extremely low occurrence, with approximately 100 cases reported so far [2]. Unlike IM and CC, ETT derives from neoplastic transformation of chorionic-type intermediate trophoblastic cells [1], [3], and is prone to be chemo-resistant [4]. Surgical intervention is recommended for ETT patients as the primary choice of treatment [4]. In clinical practice, however, due to its low incidence and limited knowledge, ETT has a high chance of being misdiagnosed and subsequently mismanaged [2], [5], [6], which may lead to progressive tumor development, metastasis, and poor prognosis [4], [7]. Therefore, an accurate diagnosis is essential to minimize the risk of mistreatment.

Pelvic ultrasound, especially transvaginal ultrasound, is the initial imaging investigation when GTN is suspected in clinical routine [8], [9]. However, little is known about the sonographic features of ETT due to its exceedingly low incidence [8]. To date, the published studies mainly focused on clinicopathological features of ETT [1], [5], [7], [10], and provided little insight into sonography [11]. The aim of this study was to identify discriminative sonographic features for the pre-operative diagnosis of ETT. We took a retrospective approach to analyze ultrasound images so as to identify distinct sonographic features between ETT and other GTNs. We also correlated these sonographic findings with histopathological features.

## Methods

We searched clinical records for all patients with ETT and PSTT from Medical Record Review System during May 2004 to December 2013 in the Women's Hospital, School of Medicine, Zhejiang University. To avoid pitfalls in the histological diagnosis of ETT and PSTT [5], we deliberately reconfirmed all the postoperative specimens of ETT and PSTT by two pathologists (B. Lu and Y. Liang) according to the WHO tumor classification guidance (2003) [12], and further verified difficult cases by a panel of antibodies including hPL, CD146, p63 etc [13], [14]. Except the cases lacking pre-surgical ultrasound documents, we finally recruited 12 ETT and 21 PSTT patients with reconfirmed pathological diagnosis. In addition, 24 IM/CC patients were matched with ETT patients on ultrasound examination date. IM patients and CC patients were clinically diagnosed according to the FIGO criteria (2000) [15], and not distinguished but combined into a single catalogue in this study, as per the common practice of gynecologists [16].

We retrieved the sonographic files of those GTN cases from Picture Archiving and Communication System. We recorded the following information: (1) location of the lesion: uterine corpus, isthmus, cervix, vagina or extra-uterine; (2) maximal diameter of the lesion; (3) morphology: solid, cystic or mixed cystic-solid; (4) border: well-defined or not; (5) Doppler signal pattern: peripheral pattern, where blood flow Doppler signals predominantly distributed at the tumor periphery, or non-peripheral pattern, where blood flow Doppler signals distributed within the boundary of the tumor, even throughout the whole tumor; (6) color score within the boundary of the tumor: from 1 to 4, according by IOTA and IETA definitions [17], [18]; (7) hemodynamic parameters of tumor vessels if measured: peak systolic velocity, end diastolic velocity, resistance index, and systolic/diastolic flow velocity ratio. The clinical data were recorded including age, parity, gravidity, presenting symptoms, FIGO stage [15], the serum human chorionic gonadotropin (hCG) level at the day of ultrasound examination, the history of GTN and treatment, antecedent pregnancy and interval time to presentation.

This is a retrospective hospital record analysis. For the purpose of clinical research, the routine clinical records of the patients in our hospital have been anonymized and the imaging data have been routinely entered into a database since 2003. Using these records for analysis in this study was approved by the Ethics Committee of Women's Hospital, School of Medicine, Zhejiang University (Reference: 20120023).

All continuous data were analyzed by student T-test. All contingency tables were assessed using the Chi-square test or Fisher's exact test. *P<*0.05 (two tailed) was considered statistically significant. All analyses were carried out using SPSS 16.0 under Windows XP (Microsoft Corporation, Redmond, WA, USA)

## Results

### Clinical presentations of ETT in comparison with other GTNs

Table 1 shows the clinical presentations of ETT and other GTNs. The age, parity, initial clinical symptom, the serum hCG level, FIGO stage were not significantly different among ETT and PSTT, IM/CC (P>0.05). The mean interval time from the antecedent pregnancy to tumor presentation of ETT was longer than that of PSTT and IM/CC (P = 0.016 and 0.002, respectively) but varied tremendously from 13 months to 11 years, which was overlapped with that range of PSTT. Antecedent pregnancy events of ETT had a greater proportion of normal pregnancy or abortion than that of IM/CC (P = 0.001), but no significant difference from that of PSTT (P = 0.103). Thus, these data show that there is no reliable clinical parameter to distinguish ETT from other GTNs.

**Table 1:** The clinical feature of ETT, PSTT and IM/CC.

		ETT (n = 12)	PSTT (n = 21)	IM/CC (n = 24)
Age		36 (26∼54)	30 (21∼44)	30 (18∼49)
Gravidity		4 (2∼6)	3 (1∼7)	3 (1∼6)
Parity		1 (0∼3)	1 (0∼3)	1 (0∼2)
Antecedent pregnancy¶	Term	6	16	2
	Abortion	4	5	2
	Hydatidiform mole	2	0	20
Interval time (month)§ ¶		71 (13∼264)	16 (4∼48)	2 (1∼8)
Initial clinical symptom	Abnormal serum hCG	4	0	15
	Irregular vaginal bleeding	3	11	4
	Amenorrhea	3	10	1
	Abnormal imaging	1	0	1
	Other symptoms	1	0	3
Serum hCG level (IU/L)^#^		1,115 (23∼177,255)	175 (1∼22,065)	11,276 (111∼84,928)
FIGO stage	I	8	19	9
	II	1	0	0
	III	1	1	15
	IV	2	1	0

Except^ #^ the values are shown in the format of “median (range)”, other continuous values are shown in the format of “mean (range)”, and counting data are shown in the format of contingency table.

§ *p*-value between ETT and PSTT is less than 0.05.

¶ *p*-value between ETT and IM/CC is less than 0.05.

Nine ETT patients had a history of chemotherapy (MTX, EMA-CO and/or EP-EMA regimen, 3-15 courses). All of them showed drug resistance to the first-line chemotherapy as witnessed by the absence of exponential decrease of serum hCG level. Two of them were shifted to hysterectomy once signs of drug-resistance appeared, while the other seven cases underwent the second-line multidrug strategy and/or radiotherapy, but recurrence occurred with a period of 6 to 66 months. Notably, 7 of 12 ETT cases were clinically misdiagnosed as IM/CC (2 cases), leiomyomas (2 cases) and ectopic pregnancy (3 cases) at the initial consultation.

### Ultrasonographic characteristics of ETT in comparison with other GTNs

Ten out of twelve ETT (83%), 21/21 PSTT (100%) and 16/24 IM/CC (67%) patients had one detectable uterine lesion in transvaginal sonographic images. One ETT patient had two lesions (one in the posterior uterine wall and the other in the uterine cervix, Figure S1 Case 10), and another ETT patient had no lesion in the uterine but metastasis in the left inguinal lymph node (Figure S1 Case 9). The remaining eight IM/CC patients (33%) had no visible lesions in the uterus.

**Figure 1:**
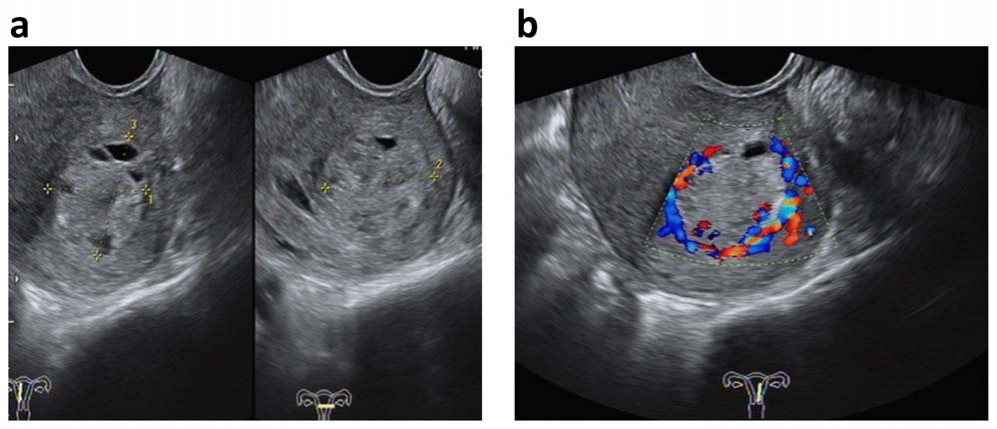
Ultrasonographic characteristics of ETT. (a) On gray-scale image, lesion was located in the left fundus of uterus, showing heterogeneously echogenic cystic-solid mass with distinct border (between cursors). (b) On Color Doppler, the abundant Doppler signals from blood flow distributed at the tumor periphery, while a few signals were showed within the boundary of tumor, which is named as “peripheral Doppler signal”.

**Figure 2:**
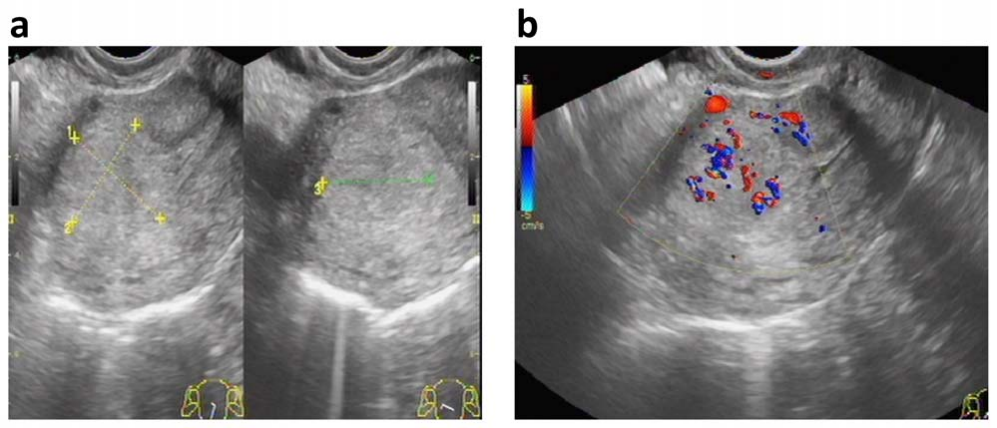
Ultrasonographic characteristics of PSTT. (a) On gray-scale image, the lesion was located in the anterior wall of uterus, showing the hyperechogenic solid mass with unclear border (between cursors). (b) On Color Doppler, the abundant Doppler signals from blood flow presented within the boundary of tumor rather than at the periphery, which is named as “non-peripheral Doppler signal”.

**Figure 3:**
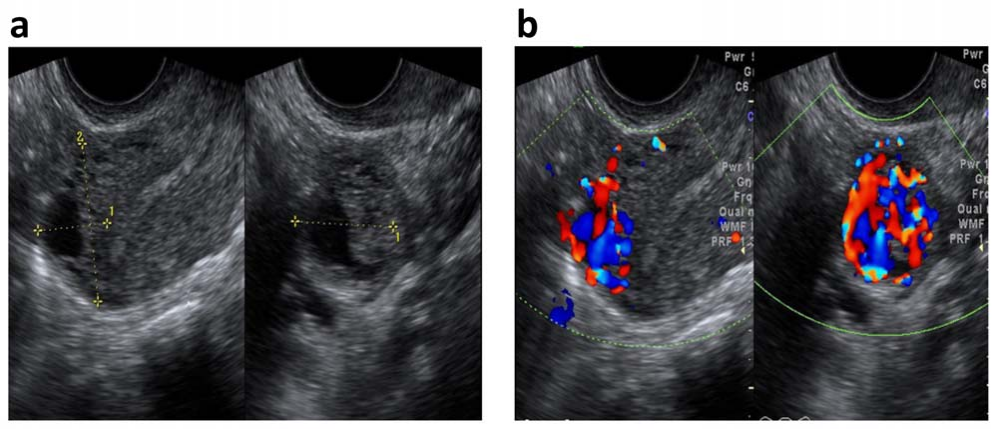
Ultrasonographic characteristics of IM. (a) On gray-scale image, the lesion was located in the uterine fundus, appearing the cystic-solid mass with unclear border (between cursors). (b) On Color Doppler image, the extremely abundant Doppler signals formed by blood flow were distributed throughout the tumor, which is named as “non-peripheral Doppler signal”. The irregular shape of cyst was the secondary arteriovernous shunts, which was fulfilled chaotic signals on color Doppler.

The ultrasonographic features of the uterine lesions among ETT, PSTT and IM/CC are listed in Table 2. More lesions located in the lower segment of uterus were observed for ETT than those for PSTT and IM/CC with the P-value of 0.005 and 0.002, respectively. On gray-scale images, ETT tended to exhibit a well-circumscribed tumor border surrounded by a hypoechogenic halo (Figure 1a and Figure S1), which was rarely observed for other two groups (both P<0.001, Figure 2a, 3a and Figure S2, S3). On Color Doppler images, ETT lesions demonstrated relatively more Doppler signal spots at the periphery than in the intratumoral area (Figure 1b and Figure S1). In contrast, both other two groups showed a non-peripheral pattern: minimal to moderate, even remarkable Doppler signal within the boundary, or throughout the tumor (Figure 2b, 3b and Figure S2, S3). The maximal diameter and all hemodynamic parameters, however, were not significantly different among ETT, PSTT and IM/CC (P>0.05).

**Table 2:** Ultrasonographic characteristics of the uterine lesion in ETT, PSTT and IM/CC.

		ETT (n = 12)	PSTT (n = 21)	IM/CC (n = 16)
Location§ ¶	Uterine corpus	6	20	16
	Lower segment of uterus	6	1	0
Maximum diameter (cm)		4.7(1.2∼10.6)	4.1 (1.1∼7.8)	3.1 (0.7∼5.7)
Morphology	Solid	7	10	3
	Cystic	0	3	0
	Cystic-solid	5	8	13
Border§ ¶	Well-defined	12	1	1
	Ill-defined	0	20	15
Doppler signal pattern§ ¶	Non-peripheral	1	21	14
	Peripheral	11	0	2
Color score* § ¶	1	7	0	0
	2	4	4	2
	3	1	9	4
	4	0	8	10
Hemodynamic parameters	PSV (cm/s)Δ	15.65 (6.20∼30.30)	27.54 (3.90∼70.40)	29.62 (20.00∼55.33)
	EDV (cm/s)Δ	8.89 (2.60∼23.70)	18.19 (1.39∼38.58)	16.97 (5.74∼38.09)
	RIΔ	0.47 (0.24∼0.69)	0.39 (0.11∼0.66)	0.43 (0.19∼0.74)
	S/DΔ	2.13 (1.28∼3.19)	1.73 (1.12∼2.85)	1.95 (1.23∼3.86)

^*^Color score 1∼4 stand for the semi-quantitative Doppler signal from absence to abundant.

ΔPSV, peak systolic velocity; EDV, end diastolic velocity; RI, resistance index; S/D, systolic/diastolic flow velocity ratio. Continuous values are shown in the format of “mean (range)”, and counting data are shown in the format of contingency table.

§ *p*-value between ETT and PSTT is less than 0.05.

¶ *p*-value between ETT and IM/CC is less than 0.05.

To further explore the formation time of the clear border with peripheral Doppler signal for ETT, we examined the time-course sonographic profiles of 2 ETT patients throughout the period of chemotherapy (3 and 5 courses, respectively). The clear border with peripheral Doppler signal was observed both pre- and post-chemotherapy for these 2 ETT patients, the same images as the 7 patients after multi-course chemotherapy and the 3 patients without treatment. These findings indicate that the clear border with peripheral Doppler signal for ETT seems like no variation with chemotherapy.

### Pathological findings of ETT in comparison with PSTT

**Figure 4:**
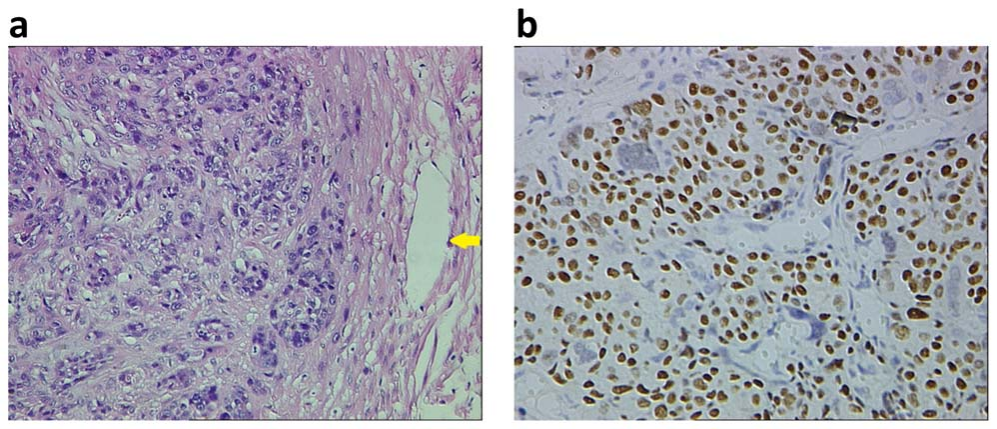
Histological features of ETT. (a) H&E staining profile showed an expansile growth pattern with pushing border, and blood vessels proximate to the tumor periphery (yellow arrows). In contrast, extensive necrosis presented within the boundary. (b) Immunohistochemical staining showed strong nuclear p63. Both images with original magnification ×200.

**Figure 5:**
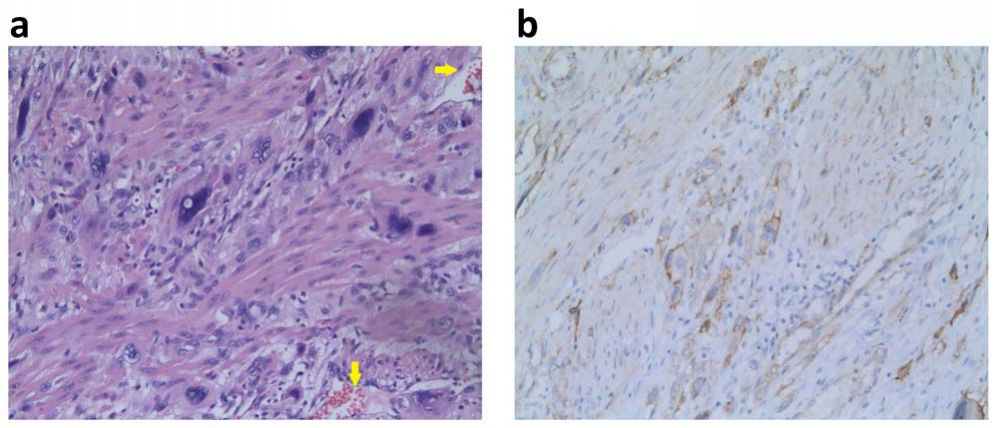
Histological features of PSTT. (a) H&E staining profile showed an infiltrating growth pattern with the penetration of tumor cells into the myometrial smooth muscle and blood vessels (yellow arrows) at the tumor periphery. (b) Immunohistochemical staining showed diffusive cytoplasmic CD146. Both images with original magnification ×200.

The pathological features of ETT and PSTT have been well described previously [2], [19]–[21]. We here addressed several major morphological differences between ETT and PSTT. All ETT cases showed a nodular, expansile pattern with a well-circumscribed pushing border without invading the surrounding myometrial muscle fibers (Figure 4a and Figure S4), whereas the cells of PSTT always infiltrated and split normal myometrial muscle fibers (Figure 5a and Figure S5). ETT was composed of a relatively uniform population of mononucleated chorion leave-type intermediate trophoblastic cells. Necrosis, typically geographic necrosis, was usually presented, and became more extensive in the non-peripheral area rather than that at the periphery. In contrast, PSTT, characterized by the proliferation of implantation site intermediate trophoblasts, showed the minimal coagulative necrosis in the focal area. Moreover, we noted a different vascular morphology between ETT and PSTT: in ETT, small vessels were located in the viable tumor cell nests, surrounded by hyalinized and necrotic materials, and more vessels at the periphery of the tumor where necrosis was inconspicuous; whereas in PSTT, tumor cells invariably invaded, migrated through the vascular walls and even replaced the vascular endothelium. Immunohistochemical staining typically showed diffuse nuclear p63, negative cytoplasmic hPL and CD146 in ETT (Figure 4b and Figure S4), while p63(-), diffusive hPL(+), and strong CD146(+) in PSTT (Figure 5b and Figure S5). Histology of IM and CC had not been reviewed because no tissue was available, since these patients responded to chemotherapy.

## Discussion

ETT, a rare type of GTN, shares similar clinical features with other GTNs except the relatively longer interval time from the antecedent pregnancy. It is prone to be clinically misdiagnosed as other GTNs and a variety of non-trophoblastic tumors. In this study, we reveal the distinct ultrasound features of ETT that can be explicitly associated with the pathological characteristics, including: (1) the well-defined border, which reflects the expansive growth pattern forming interfaces between the tumor body and the surrounding myometrial smooth muscle fibers; (2) the peripheral hypoechogenic halo, which may correlate with the dilated lymphocytic and blood vessels proximate to tumor boundary; (3) the peripheral pattern of Doppler signals can be explained by the contrasting vascular morphology between at the periphery and within the boundary. The tumor periphery presents relatively more vessels but less necrosis, whereas the more extensive necrosis within the boundary results in the far less intratumoral vascular density. Additionally, these intratumoral vessels are non-penetrated by tumor cells, thus they are too small to be detected in Color Doppler image, or be just showed as low signals formed by small volume of blood flow.

In contrast to ETT, we found that the majority of PSTT and IM/CC cases in our study showed the indistinct border and non-peripheral Doppler signal on sonographic images. PSTT and IM/CC tumors commonly exhibited an infiltrating growth pattern of tumor cells penetrating between the myometrial muscle fibers [19], [20]. This peculiar growth pattern could be one major reason why PSTT and IM/CC tumors have an indistinct border. On the other hand, the non-peripheral Doppler signal in PSTT and IM/CC can be associated with their vascular morphology. The vascular morphology changes in the region with the occurrence of PSTT or IM/CC, which includes the replacement of the vascular wall with tumor cells (placental site trophoblasts for PSTT and the chorionic villi for IM/CC), and secondary arteriovernous shunts that are common pathological change in IM [20], [21]. These reconstructive vessels are usually dilated, and thus they can be straightforwardly detected by Color Doppler, appearing the extreme blood flow signals, color aliasing and loss of vessel discreteness [9]. Such a vascular reconstruction ultimately leads to the non-peripheral Doppler signal in PSTT and IM/CC.

Our comparative results reveal that the well-circumscribed border with peripheral Doppler signal, instead of the maximal diameter and hemodynamic parameters, is a unique feature of ETT that is distinct from other GTNs. Notably, these ultrasonographic phenomena can be explained by the histopathological features. Thus, we suggest that the well-circumscribed border with peripheral Doppler signal may be a useful ultrasonographic diagnostic marker for ETT. Moreover, we find this marker appears to be persistent in ETT lesions during multiple courses of chemotherapy, implying that such a peripheral Doppler signal could be an intrinsic feature. It is meaningful for clinical practice that ETT should be suspected when the well-circumscribed border with peripheral Doppler signal appears in GTN patients with chemotherapy, particularly in patients with drug resistance. These patients might be better served by surgery rather than multidisciplinary chemotherapy. However, more clinical investigations are required to consolidate our finding based upon the limited subjects in this present study. Our multi-center study collecting a large number of samples is ongoing to validate the value of this feature in the imaging diagnosis of ETT.

## Supporting Information

Figure S1 **Ultrasound images of 12 ETT cases.** Ten out of twelve patients had one detectable uterine lesion in each case, while Case 10 had two uterine lesions and Case 9 had metastasis lesion in the inguinal lymph node. On gray-scale images, the lesions appeared heterogeneously solid or cystic-solid masses with clear border. On Color Doppler images, the relatively more Doppler signal spots formed by blood flow were distributed at the peripheral tumors, while fewer signal spots were showed within the boundary of tumors, which is named as “peripheral Doppler signal”.
(PDF)

Figure S2 **Ultrasound images of 21 PSTT cases.** Each patient had one detectable uterine lesion in ultrasound images. On gray-scale images, the lesions appeared heterogeneously solid, cystic or cystic-solid masses with unclear border. On Color Doppler images, the Doppler signal presented within the boundary of tumors rather than at the peripheries, which is named as “non-peripheral Doppler signal”.
(PDF)

Figure S3 **Ultrasound images of 24 IM/CC cases.** 16 patients had one detectable uterine lesion in each case, which showed heterogeneously solid or cystic-solid masses with unclear border on gray-scale images, and more Doppler signal spots were distributed within the boundary of tumors, or throughout the whole tumors, which is named as “non-peripheral Doppler signal”. Other 8 cases (Cases 2,3,7,8,10,12,14 and 17) had no detectable lesion in the uterus.
(PDF)

Figure S4 **Histological images of 12 ETT cases.** Cases 1, 5, 7, 10 and 11 were stained with H&E but not immunohistochemistry; Cases 3, 4, 6, 9 and 12 were stained with H&E (left) and p63 (right); Cases 2 and 8 were stained with H&E (left) and hPL (right). H&E staining profile showed an expansile growth pattern with pushing border, and extensive necrosis presented within the boundary. Immunohistochemical staining showed positive nuclear expression of p63 but negative cytoplasmic hPL.
(PDF)

Figure S5 **Histological images of 21 PSTT cases.** Cases 1, 11, 13 and 20 were stained with H&E but not immunohistochemistry; Cases 2–10, 14, 16, 18, 19 and 21 were stained with H&E (left) and hPL (right); Case 12 and 17 were stained with H&E (left) and p63 (right), Case 15 was stained with H&E (left) and CD146 (right). H&E staining profile showed an infiltrating growth pattern with the penetration of tumor cells into the myometrial smooth muscle fibers and blood vessels. Immunohistochemical staining showed positive cytoplasmic hPL and CD146, but negative nuclear p63.
(PDF)
