# Supplementary material for: A Well-Circumscribed Border with Peripheral Doppler Signal in Sonographic Image Distinguishes Epithelioid Trophoblastic Tumor from Other Gestational Trophoblastic Neoplasms
Source: PLoS One. 2014 Nov 14;9(11):e112618. doi: 10.1371/journal.pone.0112618 (PMC4232420; doi:10.1371/journal.pone.0112618)
Supplement: Figure S1 — Ultrasound images of 12 ETT cases. Ten out of twelve patients had one detectable uterine lesion in each case, while Case 10 had two uterine lesions and Case 9 had metastasis lesion in the inguinal lymph node. On gray-scale images, the lesions appeared heterogeneously solid or cystic-solid masses with clear border. On Color Doppler images, the relatively more Doppler signal spots formed by blood flow were distributed at the peripheral tumors, while fewer signal spots were showed within the boundary of tumors, which is named as “peripheral Doppler signal”. (PDF) [file pone.0112618.s001.pdf]

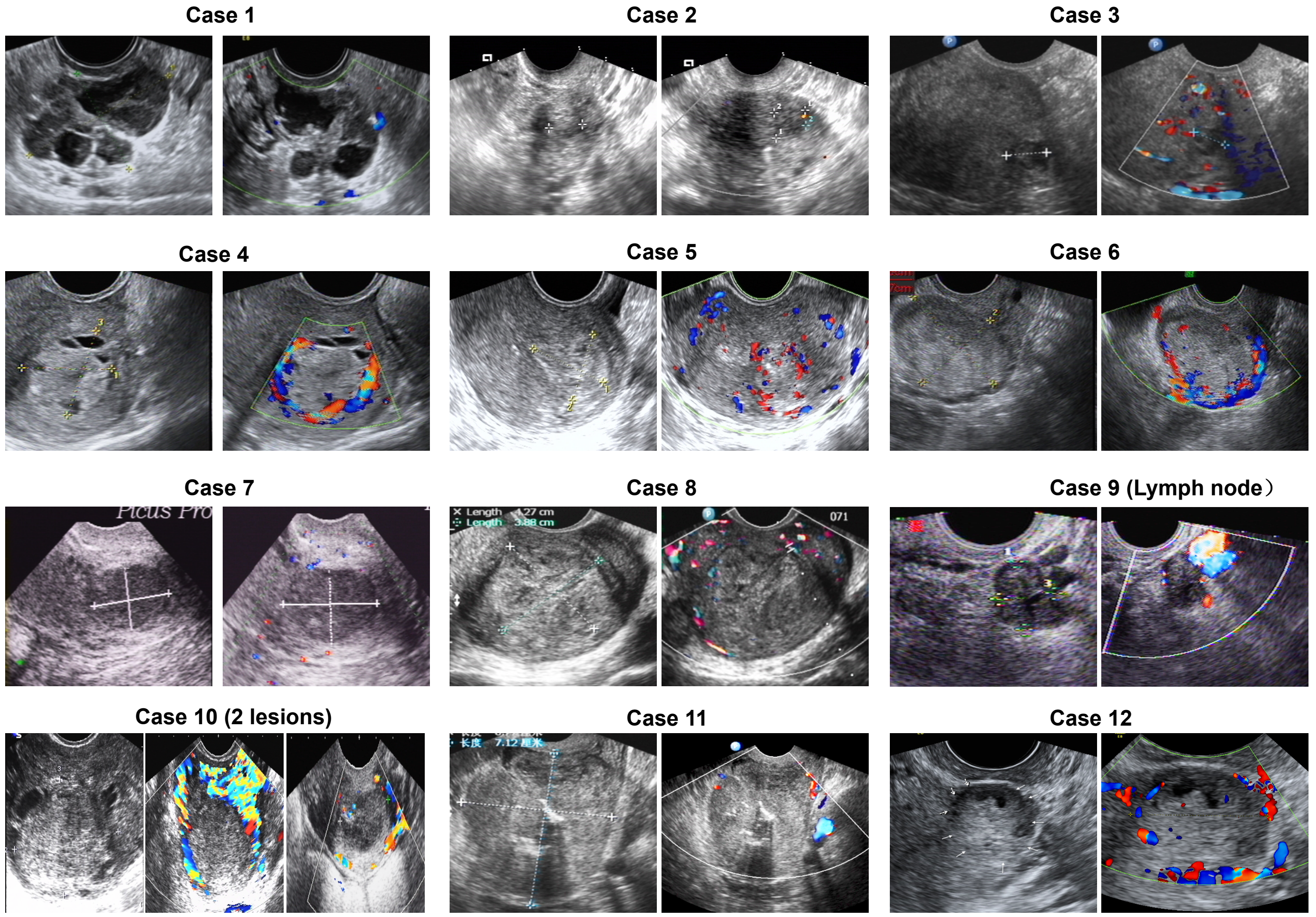

Figure S1. Ultrasound images of 12 ETT cases. Ten out of twelve patients had one detectable uterine lesion in ultrasound images, while Case 10 had two uterine lesions and Case 9 had metastasis lesion in the inguinal lymph node. On gray-scale images, the lesions appeared heterogeneously solid or cystic-solid masses with clear border. On Color Doppler images, the relatively more Doppler signal spots formed by blood flow were distributed at the peripheral tumors, while fewer signal spots were showed within the boundary of tumors, which is named as “peripheral Doppler signal”.
