# Supplementary material for: A Well-Circumscribed Border with Peripheral Doppler Signal in Sonographic Image Distinguishes Epithelioid Trophoblastic Tumor from Other Gestational Trophoblastic Neoplasms
Source: PLoS One. 2014 Nov 14;9(11):e112618. doi: 10.1371/journal.pone.0112618 (PMC4232420; doi:10.1371/journal.pone.0112618)
Supplement: Figure S2 — Ultrasound images of 21 PSTT cases. Each patient had one detectable uterine lesion in ultrasound images. On gray-scale images, the lesions appeared heterogeneously solid, cystic or cystic-solid masses with unclear border. On Color Doppler images, the Doppler signal presented within the boundary of tumors rather than at the peripheries, which is named as “non-peripheral Doppler signal”. (PDF) [file pone.0112618.s002.pdf]

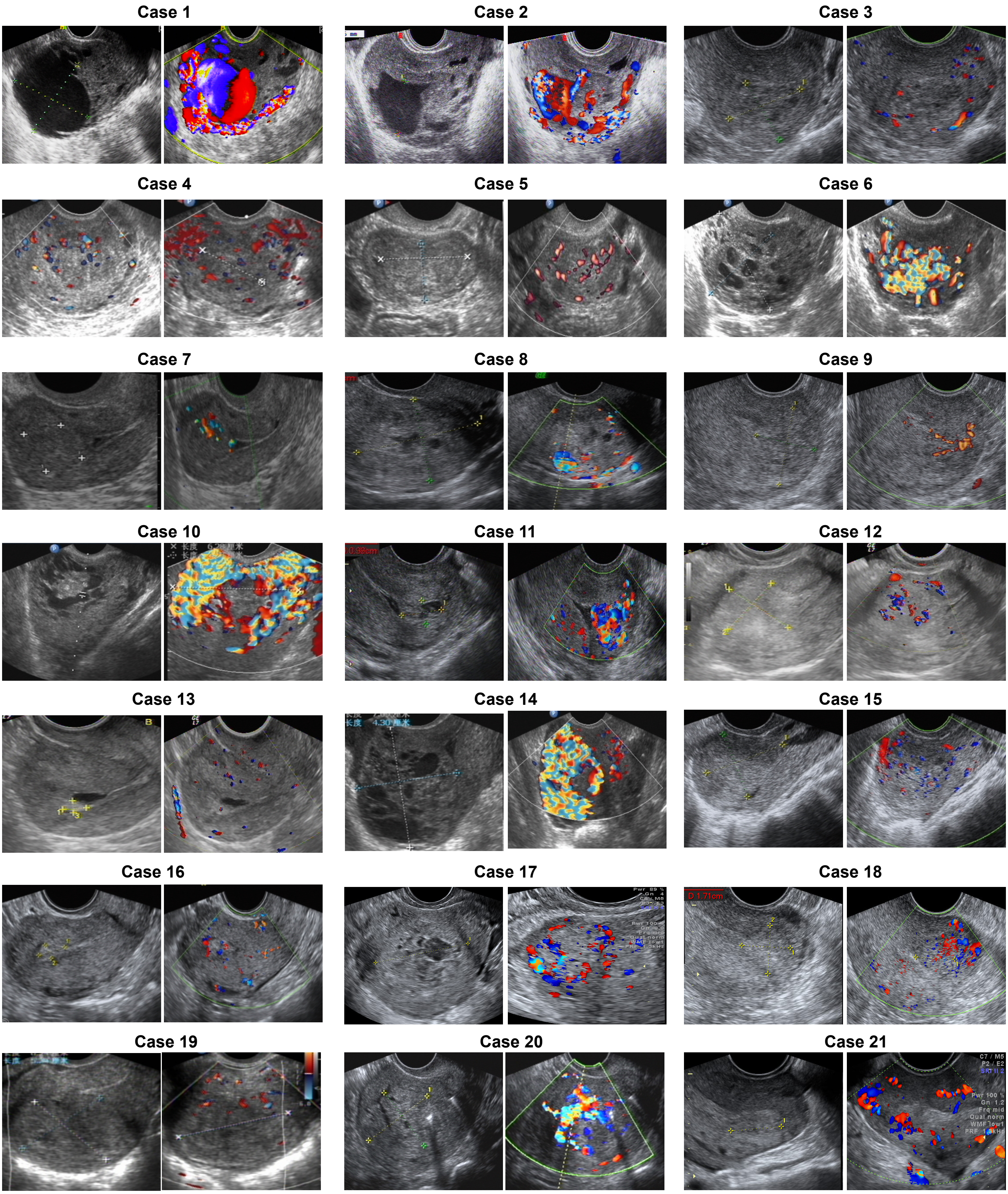

Figure S2. Ultrasound images of 21 PSTT cases. Each patient had one detectable uterine lesion in ultrasound images. On gray-scale images, the lesions appeared heterogeneously solid, cystic or cystic-solid masses with unclear border. On Color Doppler images, the Doppler signal presented within the boundary of tumors rather than at the peripheries, which is named as "non-peripheral Doppler signal".
