# Supplementary material for: A Well-Circumscribed Border with Peripheral Doppler Signal in Sonographic Image Distinguishes Epithelioid Trophoblastic Tumor from Other Gestational Trophoblastic Neoplasms
Source: PLoS One. 2014 Nov 14;9(11):e112618. doi: 10.1371/journal.pone.0112618 (PMC4232420; doi:10.1371/journal.pone.0112618)
Supplement: Figure S3 — Ultrasound images of 24 IM/CC cases. 16 patients had one detectable uterine lesion in each case, which showed heterogeneously solid or cystic-solid masses with unclear border on gray-scale images, and more Doppler signal spots were distributed within the boundary of tumors, or throughout the whole tumors, which is named as “non-peripheral Doppler signal”. Other 8 cases (Cases 2,3,7,8,10,12,14 and 17) had no detectable lesion in the uterus. (PDF) [file pone.0112618.s003.pdf]

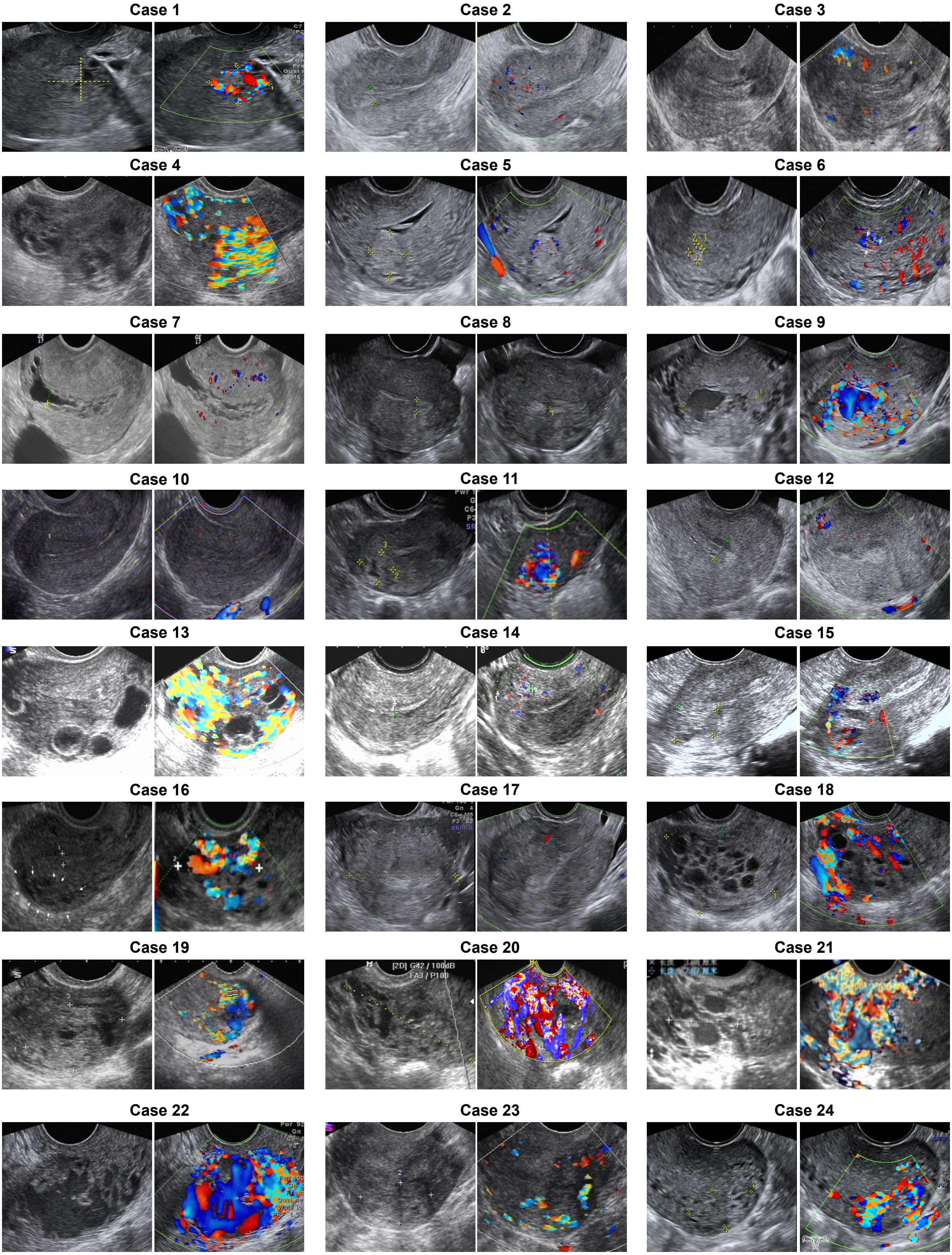

Figure S3. Ultrasound images of 24 IM/CC cases. 16 patients had one detectable uterine lesion, which showed heterogeneously solid or cystic-solid masses with unclear border on gray-scale images, and more Doppler signal spots were distributed within the boundary of tumors, or throughout the whole tumors, which

is named as “non-peripheral Doppler signal”. Other 8 cases (Cases 2,3,7,8,10,12,14 and 17) had no detectable lesion in the uterus.
