# Supplementary material for: A Well-Circumscribed Border with Peripheral Doppler Signal in Sonographic Image Distinguishes Epithelioid Trophoblastic Tumor from Other Gestational Trophoblastic Neoplasms
Source: PLoS One. 2014 Nov 14;9(11):e112618. doi: 10.1371/journal.pone.0112618 (PMC4232420; doi:10.1371/journal.pone.0112618)
Supplement: Figure S4 — Histological images of 12 ETT cases. Cases 1, 5, 7, 10 and 11 were stained with H&E but not immunohistochemistry; Cases 3, 4, 6, 9 and 12 were stained with H&E (left) and p63 (right); Cases 2 and 8 were stained with H&E (left) and hPL (right). H&E staining profile showed an expansile growth pattern with pushing border, and extensive necrosis presented within the boundary. Immunohistochemical staining showed positive nuclear expression of p63 but negative cytoplasmic hPL. (PDF) [file pone.0112618.s004.pdf]

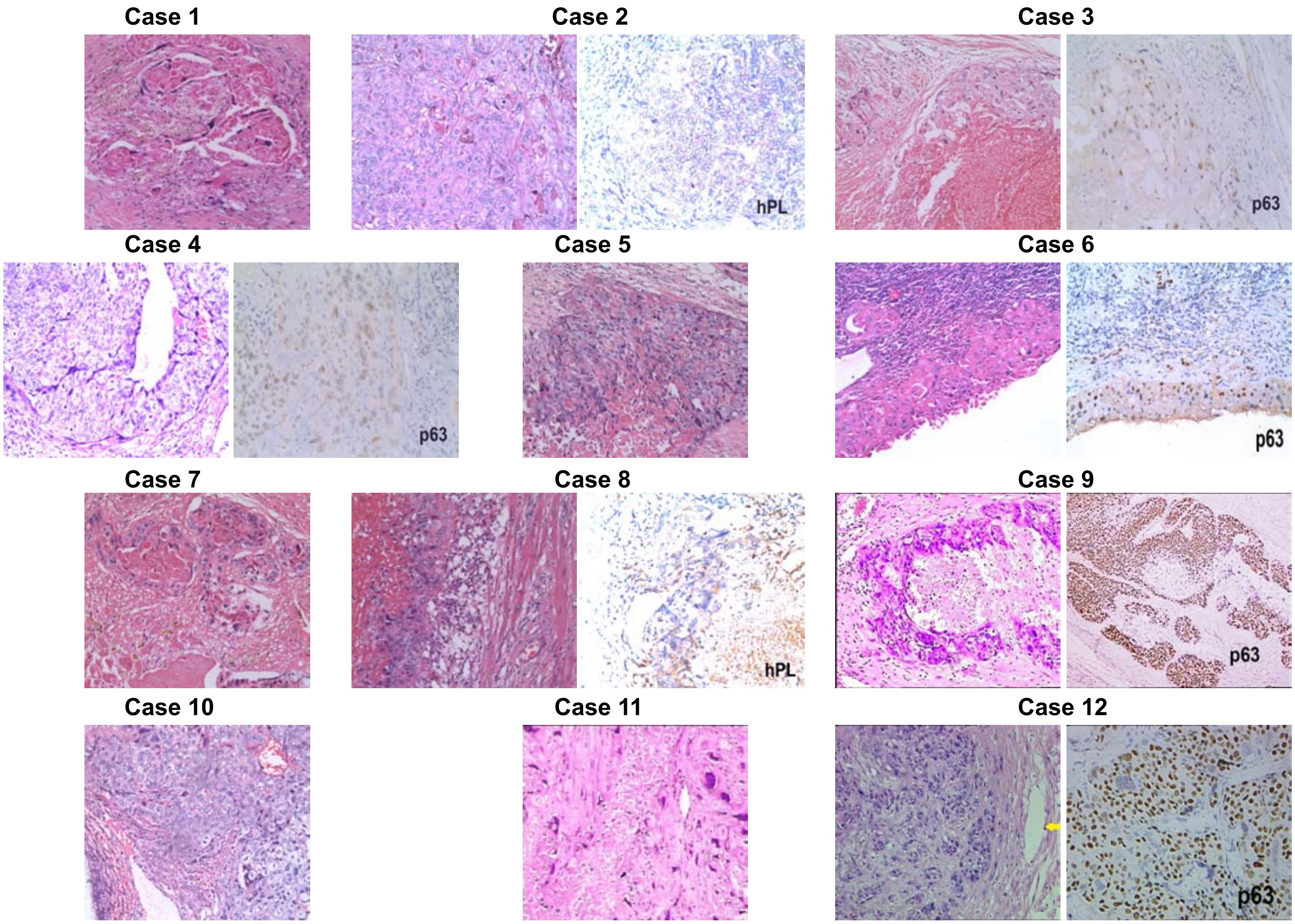

Figure S4. Histological images of 12 ETT cases. Cases 1, 5, 7, 10 and 11 were stained with H&E but not immunohistochemistry; Cases 3, 4, 6, 9 and 12 were stained with H&E (left) and p63 (right); Cases 2 and 8 were stained with H&E (left) and hPL (right). H&E staining profile showed an expansile growth pattern with pushing border, and extensive necrosis presented within the boundary. Immunohistochemical staining showed positive nuclear expression of p63 but negative cytoplasmic hPL.
