# Supplementary material for: A Well-Circumscribed Border with Peripheral Doppler Signal in Sonographic Image Distinguishes Epithelioid Trophoblastic Tumor from Other Gestational Trophoblastic Neoplasms
Source: PLoS One. 2014 Nov 14;9(11):e112618. doi: 10.1371/journal.pone.0112618 (PMC4232420; doi:10.1371/journal.pone.0112618)
Supplement: Figure S5 — Histological images of 21 PSTT cases. Cases 1, 11, 13 and 20 were stained with H&E but not immunohistochemistry; Cases 2–10, 14, 16, 18, 19 and 21 were stained with H&E (left) and hPL (right); Case 12 and 17 were stained with H&E (left) and p63 (right), Case 15 was stained with H&E (left) and CD146 (right). H&E staining profile showed an infiltrating growth pattern with the penetration of tumor cells into the myometrial smooth muscle fibers and blood vessels. Immunohistochemical staining showed positive cytoplasmic hPL and CD146, but negative nuclear p63. (PDF) [file pone.0112618.s005.pdf]

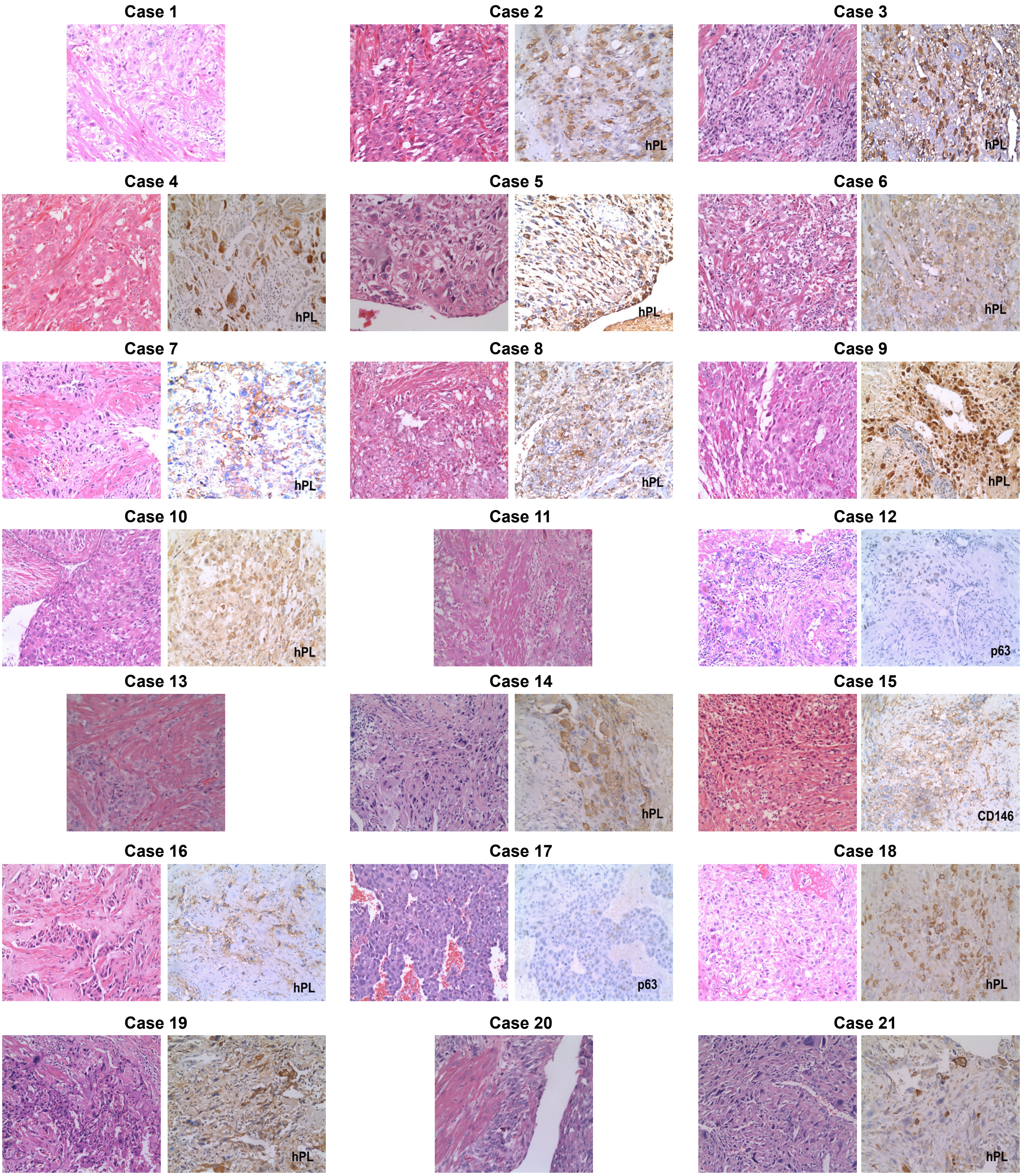

Figure S5. Histological images of 21 PSTT cases. Cases 1, 11, 13 and 20 were stained with H&E but not immunohistochemistry; Cases 2-10, 14, 16, 18, 19 and 21 were stained with H&E (left) and hPL (right); Case 12 and 17 were stained with H&E (left) and p63 (right), Case 15 was stained with H&E (left) and CD146 (right). H&E staining profile showed an infiltrating growth pattern with the penetration of tumor cells into the myometrial smooth muscle fibers and blood vessels. Immunohistochemical staining showed positive cytoplasmic hPL and CD146, but negative nuclear p63.
